# Mothers’ Perceptions of Toddler Beverages

**DOI:** 10.3390/nu10030374

**Published:** 2018-03-19

**Authors:** Manuela Rigo, Jane Willcox, Alison Spence, Anthony Worsley

**Affiliations:** School of Exercise and Nutrition Sciences, Deakin University, 221 Burwood Highway, Burwood, VIC 3125, Australia; m.rigo@deakin.edu.au (M.R.); jwillcoxresearch @gmail.com (J.W.); a.spence@deakin.edu.au (A.S.)

**Keywords:** toddlers, preschoolers, mothers, parents, sugar-sweetened beverages, Repertory Grid Technique, Laddering Technique, qualitative methods, Australia

## Abstract

Background: The prevalence of obesity among Australian pre-school children is a major concern with links to poor health outcomes. One contributing factor is excess energy intake. Sugar-sweetened beverages are energy-dense, nutrient-poor, readily available and have been implicated in the increasing prevalence of obesity. Furthermore, preschooler beverage consumption may develop into dietary habits that track into adulthood. There is little research on factors influencing parents’ decision-making when serving beverages to their preschoolers, or on mothers’ perceptions of preschooler’s beverages. The aim of this study was to explore mothers’ perceptions of commonly consumed preschooler beverages. Methods: The Repertory Grid Technique and the Laddering Technique methodologies were utilized in interviews with 28 mothers from Melbourne, Australia, to explore beverage perceptions. Results: A large number of diverse perceptual categories (‘constructs’) (*n* = 22) about beverages were elicited, demonstrating the complexity of mothers’ perceptions when making beverage choices for their preschoolers. The five most common categories were related to health, sugar, dairy, packaging, and additives. Thematic analysis of responses from the laddering method identified three major themes: concerns about the types of beverages mothers would like to provide their preschoolers, the healthiness of a beverage, and the sugar content. Conclusions: Mothers’ perceptions of beverages are sophisticated and need to be included in the design of health communication strategies by health promoters and government agencies to influence mothers’ beverage selections for their preschoolers.

## 1. Introduction

The high prevalence of overweight and obesity among young children is a major public health concern and is linked to poor health outcomes in childhood and adulthood, including non-communicable diseases such as Type 2 diabetes [1,2], sleep apnoea [3], and adverse mental health outcomes [4]. Poor diet quality, including excess energy, is associated with overweight and obesity in children including preschoolers [5]. Frequent and energy-dense, nutrient-poor beverage choices are indicators of overall poor diet quality [6], that may lead to excess energy intake and hence contribute to overweight and obesity. In addition, there is consistent evidence that dietary habits established in childhood track into adulthood [7,8]. Excess weight gain is difficult to reverse with interventions, thus early prevention is recommended [9,10].

Mothers are generally considered as the primary household gatekeepers [11]. They are typically primarily responsible for bringing beverages into the household and thus control their availability and accessibility [12]. They also influence beverage consumption through role modelling what, when and how to drink [13,14].

Mothers’ perceptions or beliefs are likely to influence the types of beverages they provide for their preschoolers [15]. Unfortunately, there have been relatively few studies of mothers’ perceptions of the beverages they provide for their preschoolers (or ‘toddlers’), aged two to four years. Seven studies have examined mothers’ perceptions of young children’s beverages but four of them focused only on one attribute, health [16,17,18,19]. Two of the remaining three studies examined several attributes of beverages [20,21], although one of these examined only one beverage, chocolate-flavoured milk on six attributes [20]. In both these studies the researchers nominated the beverage characteristics that the respondents rated. Only one study in this area did not pre-empt mothers’ perceptions of preschoolers’ beverages and allowed them to identify attributes important to them [22]. 

More broadly, two studies of women’s fruit and manufactured snack perceptions [23,24] are relevant here as they allowed participants to nominate their own food perceptions [25]. Both utilised the Repertory Grid Technique (RGT) to elicit perceptions of snacks compared to manufactured snack foods. Jack et al. [23] examined the self-elicited perceptions and the context of consumption of 15 snack foods, including sweet, savoury, fresh and processed in a sample of 12 friends and family members. These perceptions where then developed into a questionnaire which was administered to a sample of 51 women, aged 21–55 years, employed at a college in Edinburgh. They found that the women held a wide variety of perceptions, but these could be grouped into convenience and indulgence for manufactured snacks, whereas fruit was seen to be more suitable in certain contexts, e.g., for breakfast. Weston [24] examined mothers’ perceptions, from 12 mothers, of six fruits and six manufactured, energy-dense and nutrient-poor, snacks commonly served to children aged two to five years in Australia. Again, a wide variety of perceptions were elicited. Subsequently these were incorporated in a questionnaire which was administered to 238 preschoolers’ mothers. The results showed that mothers had four goals in mind when they fed snacks to their preschoolers: convenience, satiety, eating nutritious food and freedom from additives. 

The present study was underpinned by the theoretical framework of the Food-Related Lifestyle Model [15]. This framework links lifestyle dimensions, such as beliefs about the concrete aspects of food and beverages (e.g., appearance, taste), higher order or quality attributed (e.g., nutritiousness), perceived consequences of consumption (e.g., health or mood effects), procedural scripts (shopping or meal-preparation skills), and personal values to food and beverage selection. The model has been validated in several countries and in cross-cultural settings [26,27,28].

In summary, there is a gap in the research literature in regards to mothers’ perceptions of preschoolers’ beverages. Therefore, the overall aim of this study was to investigate the beliefs and perceptions of commonly available preschoolers’ beverages held by mothers in Melbourne, Australia.

## 2. Materials and Methods

### 2.1. Study Design

Two distinct methods were used in this study to provide a rich, in-depth exploration of the topic. The first method used was the RGT which is an established method based on Personal Construct Theory (PCT) [25,29,30,31,32,33]. PCT proposes that people understand or make sense of their world through their perceptions [30,32,33]. RGT is a semi-qualitative approach in which participants are asked multiple times to compare and contrast random sets of three ‘elements’ (in the present case: beverages), allowing the elicitation of bipolar ‘constructs’ (beliefs or perceptions) from the participant’s reality [25,30,32], rather than the views of the researcher [34,35] .

The second method used in this study was “Laddering” [36]. It is based on means-end-chain theory [37] and allows the exploration of participants’ motivations that underlie their beliefs or perceptions of objects such as children’s beverages. It provides the link between the product attributes and the benefits of consumption and values of the consumer [36].

### 2.2. Study Participants

Mothers of children aged two to four years residing in two geographical areas in Melbourne, Australia were invited to participate in the study. The two areas were purposefully selected to represent different levels of socio-economic status according to the Australian Bureau of Statistics’ Socio-Economic Indexes for Areas (SEIFA) [38]. We aimed to recruit approximately 15–20 participants based on the numbers involved in the qualitative portion of the study by Jack et al. [23] and Weston [24] of 12 participants. Flyers promoting the study were e-mailed to organizations that catered to young children, such as local swimming pools, crèches and libraries, to display to members. The senior author (MR) also visited some of these organizations to distribute the flyers in person. From details on the flyer, interested participants could contact the researcher via phone or e-mail for further information and a consent form. Mothers could arrange an interview at a time convenient to them and child and bring their children with them. Inclusion criteria included mothers with children aged two to four years and sufficient English to read the plain language statement. All participants received a $15 supermarket voucher in appreciation of their time, along with a unique summary of their perceptions derived from WebGrid Plus [39].

Ethics approval was obtained from the Human Research Ethics Committee at Deakin University, on the 10 April 2017, reference HEAG-H 35_2017. 

### 2.3. Procedure

Consenting participants were invited to attend an interview at the recruitment venue or at Deakin University, and in-depth interviews were conducted. To review and streamline the procedure eight pilot interviews with mothers of preschoolers were conducted. These data were not used in the final analyses.

Each of the interviews were divided into two sections involving the administration of the RGT and then the Laddering Technique for the constructs that the participants considered important to them. Participant’s socio-demographic characteristics were collected using a short questionnaire at the conclusion of the interview. These included maternal age, educational background, occupation, postcode, ethnicity, marital status, and whether the participant was the main shopper. 

To determine the mothers’ perceptions of children’s’ beverages, thirteen images of drinks commonly consumed by Australian preschoolers in the 2011–2012 National Nutrition and Physical Activity Survey (NNPAS) [40] were selected. Images were selected to represent a wide variety of products including different costs, nutritional values, and packaging types, with two featuring animated characters, given the appeal of animated characters to young children [41,42]. The interviews were audio-recorded and transcribed. 

### 2.4. The Repertory Grid Technique

**Figure 1:**
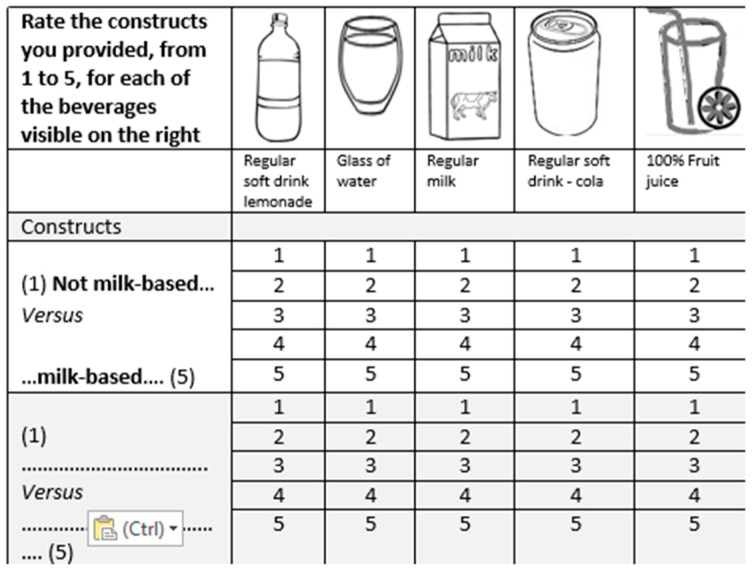
A portion of the bipolar scale on which constructs were recorded. Images have been changed to sketches to protect copyright.

The beverage images were printed on A4 sized paper and presented in sets of three (triads). Mothers were asked which beverage was different from the other two and why. The mothers were advised that there were no incorrect or correct responses and that the researchers were only interested in the mother’s own perceptions. The response to each triad (termed ‘constructs’ in PCT) were recorded in written form on five-point bi-polar scales (Figure 1). In judging the triads, the only caveat was that the participant could only use a construct once. Up to 12 sets of randomly pre-selected triads were presented to the mothers in the same order. Participants continued to judge new triads while they could provide a unique construct or until they had viewed all twelve triads. Once all the constructs had been elicited the mothers were asked to rate all 13 beverages according to the constructs that they had provided. For example, a construct might be ‘milk-based versus non milk-based’; subsequently all the beverages would be rated on a five-point bi-polar scale with one anchor point being ‘not milk-based’ (scored as 1) and the opposite anchor point being ‘most milk-based’ (scored as 5). 

### 2.5. The Laddering Technique

In the second part of the interview, the participants were asked if each of the constructs they had provided was important to them, and if so, why? Once the participant had provided a response, she was asked ‘why?’ again for further clarification. The laddering technique is qualitative in nature and the why questions are open-ended to explore the underlying motivations and values of the participants. Those participants who answered “no” to any constructs, i.e., they did not think it was important to them, were not asked the laddering questions for those constructs.

### 2.6. Data Analysis

Descriptive statistics were used to summarize the participants’ socio-demographic characteristics (Table 1).

### 2.7. Categorisation of Constructs

The constructs were grouped into similar categories according to the frequency of their key words (Table 2) [43,44]. The research team discussed and agreed on the categorization of these constructs.

### 2.8. Analysis of Mothers’ Repertory Grid Data

Each mother’s ratings of all the beverages were analysed using WebGrid Plus [39]. In this program, participants’ data were analysed by principal components analysis to derive ‘perceptual maps’ of each mother’s perceptions of the beverages [31,45].

**Figure 2:**
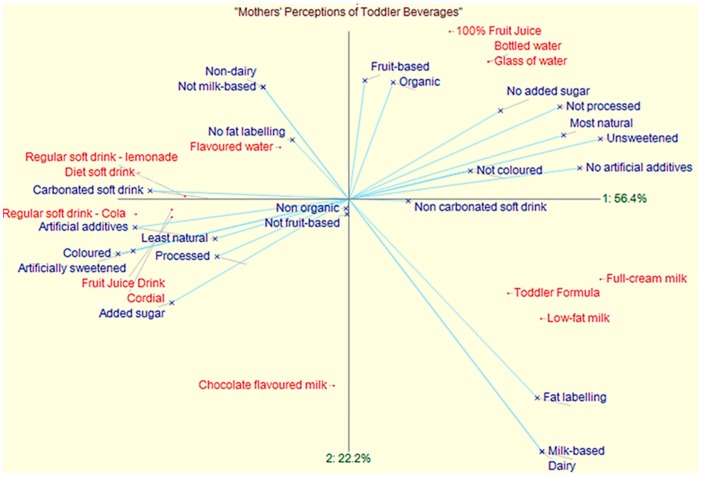
An example of a conceptual map of intermediate complexity.

In the resulting perceptual maps, beverages that are in close proximity to a construct indicate that the participant perceived the beverage as having that particular attribute. Conversely, beverages that are far from a construct tend not to possess that construct in the mother’s mind (Figure 2).

### 2.9. Thematic Analysis of the Statements Derived from the Laddering Technique

**Figure 3:**
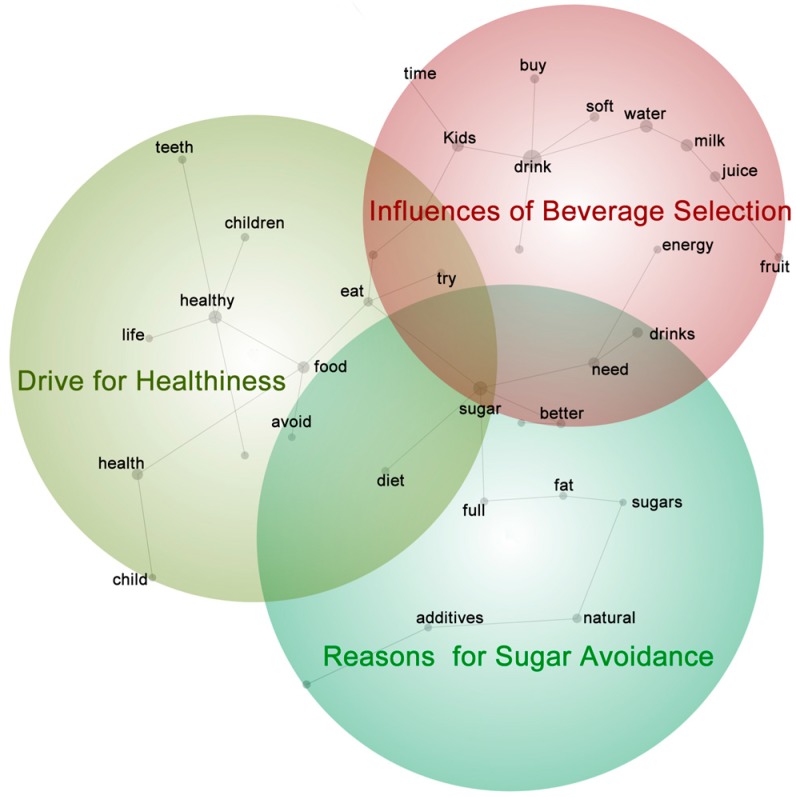
The themes derived from the laddering process. Theme size is set to 70%.

The mothers’ reasons for the importance of specific constructs, obtained from the laddering technique, were analysed via thematic analysis [34,35]. The statements were entered into Leximancer, an automatic thematic analysis software package. Leximancer uses statistical algorithms based on non-linear dynamics and machine learning to identify and group words or phrases according to their frequency and their co-occurrence [46]. These are termed ‘concepts’. Concepts that cluster together form ‘themes’. The themes are “heat mapped” according to the light spectrum; the red circle represents the strongest theme, and blue/violet is the weakest theme (Figure 3).

## 3. Results

Twenty-eight mothers of preschoolers aged two to four years participated in the interviews. Six further women who had expressed interest declined to participate for varying reasons (e.g., time constraints), and their demographics were not collected. The duration of the interviews was 20–55 min, with an average of 37 min. 

The majority of the mothers (25/28) were aged over 30 years and all but one were married or in a de-facto relationship. Over two thirds (19/28) had a bachelor’s degree or higher and 61% (17/28) were born in Australia with the remaining from ten different countries. Over four fifths (23/28) were primarily responsible for the procurement of foods for the household, and a further four participants shared this responsibility. Although mothers were recruited within organizations in the targeted area some lived in neighbouring suburbs of differing socio-economic position (SEP) based onSEIFA [38]. In total 17 mothers were in the high SEP, just over one fifth (6/28) were in the mid-SEP, and five were in the low SEP. 

The number of constructs elicited per participant ranged from five to 12. The total, 28 participants provided a diverse range of 312 constructs about preschoolers’ beverages. These were grouped into 22 categories (Table 2). The six most frequently mentioned perceptual categories accounted for 54% of all the constructs. Health and its sub-concepts, hyperactivity, nutrition, satiety, digestion, and oral health, were most frequently mentioned. In second place was sugar, in all its forms, from natural and artificial, intrinsic and extrinsic. The remaining top four construct categories were dairy, packaging type, and the presence or absence of ‘additives’. 

### 3.1. Perceptual Maps of Mothers’ Constructs

Each mother’s repertory grid data yielded a unique perceptual map of their constructs of the 13 beverages. There was great diversity within and between the maps. One example of a map of intermediate complexity is shown in Figure 2. Details of the other maps are available from the senior author. This mother perceived fruit juice drink and cordial as having similar attributes and she saw them low in “naturalness”, processed, coloured and artificially sweetened. 

### 3.2. Themes Derived from the Laddering Responses

Three main themes were identified, named as: ‘Influences of beverage selection’, ‘Drive for healthiness’ and ‘Reasons for sugar avoidance’ (Figure 2). Six minor themes included ‘fruit’, ‘natural’, ‘body’, ‘teeth’, ‘time’ and ‘child’. There was some overlap of themes, for example many mothers mentioned ‘sugar’ and ‘drink’ in the same sentence. 

#### 3.2.1. Theme 1: Influences of Beverage Selection

The mothers were particularly concerned about the drinks their preschoolers consumed. All mothers considered certain beverages necessary or ideal for preschoolers such as water, milk and some mentioned juice, and they reasoned that these choices were important for health.

“*The milk and some orange, like a fruit juice, they good because, and their taking. They need a water a little bit, but the cokes and other thing they not good for kids. Water is most important for them*.”

ID: 110, low SEP

There was also consideration of the purpose of the beverage by three mothers, of whether it quenched thirst or warded off hunger. Four mothers recognised that their children had limited appetites, or sometimes no appetite, and if a caloric beverage was provided, the child would not be able to consume a more nutritious food or beverage.

Four mothers mentioned their children’s preferences, but most mothers decided they knew best. In an attempt to control consumption of undesirable beverages four mothers would ensure the beverage was inaccessible, for example supermarket shopping without her pre-schooler.

“*I don’t want the kids drink much soft drink although they really like it. So like if I take them to the supermarket and they see it and want to buy it. If I don’t buy it they will not drink it. Usually I don’t take them to the supermarket*.”

ID: 117, high SEP

In contrast, two mothers mentioned that their children influenced the beverage they received through their behavior.

“*If she does not get juice when she asks for juice then the tantrum is not worth it*.”

ID: 106, high SEP

Eleven mothers acknowledged that the social and food context were considered when selecting a beverage for preschoolers. Therefore, a sugar-sweetened beverage might not be suitable for breakfast but acceptable for a birthday celebration.

“*When we go to McDonalds we buy these things (pop-top fruit drink or soft drink). If we have a birthday or something*.”

ID: 127, low SEP

#### 3.2.2. Theme 2: Drive for Healthiness

All the mothers wanted their children to have a good, disease-free, physically active and emotionally balanced life. Most acknowledged that both food and drink were important for good health, which included oral health, being physically fit and having strong disease immunity. Four mothers commented that it was important to develop habits that achieve healthy outcomes.

“*I want him to grow healthy. I am trying to do everything I can in my power to make sure he has a healthy, well-balanced diet, to grow up healthy, and physically fit*.”

ID: 122, high SEP

Half of the mothers were also concerned about avoiding certain substances, like additives, or caffeine for health reasons, and the unknown consequences of these items.

“*…because the long-term effect of those additives on health are not always known and I think especially for young kids you don’t want really want them consuming these artificial additives if you’re not really sure how it will affect them short or long-term*.”

ID: 105, low SEP

#### 3.2.3. Theme 3: ‘Reasons for Sugar Avoidance’

Twenty-six mothers were concerned about sugar in their preschooler’s diet. Generally, they felt their preschoolers consumed enough sugar in food, so additional sugar from drinks was excessive. One mother was concerned that sugar had worse consequences than fat in food.

“*I think particularly for drinks, it should, it is always one of those areas where if you are going to cut down on sugar just not having sugary drinks is the one of the easiest ways to do that*”

ID: 101, high SEP

Twenty-four of the mothers also worried about the short and long-term effects of sugary beverages causing conditions like hyperactivity, tooth decay, and diabetes. In contrast, two mothers believed that sugar was important and that it had a place in a child’s diet.

“*I think that sugar provides something to the body. I am not sure because I am not a doctor but I think that sugar still provides something to us, we need maybe a little bit because if we use too much we may be diabetes later. If you don’t have sugar you will lack the nutrition or something like that*.”

ID: 117, low SEP

Three mothers differentiated between different types of sugars, for example: natural, added and artificial sugars.

“*I prefer natural sugar because it is natural, you still have to keep track of how much she’s having, but at least if it is natural it is better for you*.”

ID: 115, high SEP

## 4. Discussion

This study combined two methodologies, RGT and Laddering Technique, to provide rich data and in-depth exploration of mothers’ perceptions of preschoolers’ beverages. This is in contrast to most previous studies that have only focused on one attribute, health [16,17,18,19]. This study identified many unique and diverse perceptions of beverages commonly served to young children and also identified some of the motivations underlying their purchase and consumption.

The utility of the Food Related Lifestyle Model’s was generally supported by the elicited perceptions which were complex and multi-faceted. For example, mothers mentioned their preference for serving milk over formula because of its convenience, a higher order attribute. Mentions of their preference for organic beverages because of their naturalness represent another higher order quality factor. References to lids on beverages are examples of concrete attributes that were linked to freshness and convenience (higher order quality factors). Similarly, references to the calcium content of milk can be seen as a concrete attribute but also as part of a shopping script: knowing and checking which beverages provided this nutrient. Finally, the avoidance of sugar-sweetened beverages to avoid child hyperactivity is a good example of a perceived consequence. 

### Multiple, Diverse, Perceptual Constructs

Although perceptual maps have been derived from previous studies of food perceptions [23,47] and preschoolers’ snacks [24], to the best of the authors’ knowledge, no other studies have used the RGT to examine mothers’ perceptions of preschoolers’ beverages. The perceptual maps of these beverages were unique to individual mothers and ranged from being very simple to highly complex. Nevertheless, almost 70% of all the constructs could be grouped into ten major categories, ranging from extrinsic concrete attributes, e.g., ‘packaging’, to abstract higher-order attributes, e.g. ‘nutrition’ and ‘marketing to children’.

Some of the perceptual categories shown in Table 2 have been reported before. These include the various health-related constructs like hyperactivity, nutrition, satiety, digestion, dental health [16,21,22]; the mainly negative views of sugar and sugary beverages [16,17,20,22,48]; the benefits of milk [49], particularly for breakfast and at bedtime [21]; the concrete attributes of packaging such as lids to prevent spillage [19,22]; and the fear of additives [16,19]. The most novel feature of this study is the sheer diversity of the perceptions held by this small group of mothers and the complexity of the combinations of perceptions (‘constructs’) held by individual mothers. Whilst health related constructs were predominant, other aspects of beverages were also mentioned, such as their naturalness, content, appearance and suitability for children. Therefore, it cannot be assumed that one or two beverage attributes are important to mothers; intensive elicitation approaches are required to identify the main perceptions held within a population group.

The three major motivational themes derived from the thematic analysis of the laddering responses are consistent with previous research. The ‘Influences on Beverage Selection’ theme is about the contexts and maternal strategies that affect beverage purchasing and the control of the child’s behavior by the mother or the child. These include the use of sugar-sweetened beverages as treats, the avoidance of situations such as supermarkets in which clashes between the mother’s and child’s purchase wishes might occur (‘pester power’) [50,51,52,53,54,55], and the provision of beverages such as sugar-sweetened beverages so mothers could avoid their tantrums (also reported in previous work [21,22]). This is similar to Weston’s [24] findings that satisfying the child’s demands and appetite were major considerations for mothers. 

The ‘Drive for Healthiness’ theme highlights the nutritional trade-offs mothers may make between different beverages and foods according to their nutritional properties, for example, providing a chocolate-flavoured milk for its calcium content despite its sugar and additives. The theme is also linked to the development of healthy beverage preferences and life-long healthy habits [56,57,58,59]. Mother’ beliefs in the importance of providing healthy drinks and setting up healthy habits concur with previous research that has shown that the early years are critical for gaining knowledge of which foods (and beverages) are suitable to eat (and drink) [55]. This highlights the importance of this research as mothers may provide a beverage to their preschoolers based on their perception of healthiness, but the beverage may not be recommended by dietary guidelines. Regularly supplying this beverage to the child may influence the child’s preference and develop a habit or a ‘junk food’ dietary pattern with an excess of discretionary foods and beverages [6,57].

The ‘Reasons for Sugar Avoidance’ theme is topical, with over 200 articles in the “Newsbank” database of Australian newspapers [60] listed in the past 12 months and globally there has been an introduction of a sugar-tax on sugar-sweetened beverages in many countries [61,62]. The introduction of this tax has also been recommended by the Australian Medical Association [63]. This suggests that the perceptions identified in this study reflect recent shifts in public consciousness towards the dangers of excessive sugar consumption. This is a theme that is promoted by industry with increased production of low-sugar soft drinks [64] and health professionals, e.g., the dietary guidelines [65]. The increasing evidence of the effect of sugar-sweetened beverage consumption on the rising level of obesity in children [66,67] underscores the need for regular research in this area to gain insights on current beliefs that influence beverage consumption. 

Some products’ attributes such as taste and financial cost have been reported to be primary influences over adult food [67,68,69,70] and beverage selection [22,71,72] in many consumer research studies, but these were rarely mentioned by the mothers in the present study. Clearly, there are likely to be many differences between the context of this study of mothers’ perceptions of preschoolers’ beverages and adult food selection. As the laddering findings show, there are several higher order motivations underlying the mothers’ selection of beverages for their children. Furthermore, the women in this study were relatively well educated and the majority were from mid to high SEP, which is associated with better diet quality [73,74] and fewer monetary constraints [75]. 

The present findings clearly show that preschoolers’ mothers hold a great variety of perceptions about beverage attributes. When researchers pre-select certain perceptual attributes with little empirical foundation (e.g., cost and taste are commonly used), these may not reflect the diversity or priorities of perceptions and beliefs of the target population [76]. Any health promotion strategies, whether by health promoters, researchers or companies, should consider elicitation of perceptions as a first step. Such strategies are likely to be more effective if there is an understanding of the collective consciousness and individual interests of the target audience [77]. This would allow the creation of more tailored, and hopefully more effective, [78] health-focused strategies, consistent with the values and beliefs that are salient to the target population. 

A limitation of the study was that the mothers were relatively well-educated and generally of high SEP so the findings may not reflect the socio-economic diversity of the Melbourne population. For example, mothers of low SEP may have perceived cost to be more important. However, the participants were ethnically diverse with 11 nationalities being represented and this allowed a broad range of perceptions to be elicited. The large number and variety of constructs identified in the study suggests that many perceptions of preschoolers’ beverages important to the mothers were found. It remains for future research to examine the likely influences on these perceptions, for example, children’s responses to beverages and mother’s own preferences, and the extent to which they influence preschoolers’ dietary behaviours. 

## 5. Conclusions

Twenty-two categories of mothers’ perceptions of young children’s beverages were identified, indicating that mothers hold multiple, diverse perceptions. The perceptions ranged from ‘concrete’ attributes, e.g., packaging, to more abstract ‘higher-order’ perceptions, e.g., nutrition. These perceptions reflect the utility of the Food Related Lifestyle Model [15]. Thematic analysis revealed that the main motivational concerns for mothers were whether the type of beverage was suitable for their child in terms of context and behavioural control; the healthiness of the product in terms of nutritional trade-offs and long-term health habit formation; and the sugar content of the beverages. Future interventions to influence beverage consumption or research on beverage purchasing and perceptions and beliefs should include a perception elicitation component to determine the salient issues relevant to the target population. The findings could also inform interventions, planned for the near future, that educate parents of preschoolers about ways to determine the sugar concentration of beverages in order to make healthy choices.

## Figures and Tables

**Table 1:** Descriptive demographics of the sample.

Mothers	28
Age (years)	
25–29	3
30–34	8
35–39	12
≥40	5
Marital Status	
Married/De facto	27
Single/Divorced	1
Education	
Year 12 equivalent	4
TAFE ^1^ or trade qualification	5
University Degree or higher	19
Ethnicity	
Australian-born	17
Other nationality	11
Main Shopper in the household	
Yes	23
Shared the responsibility	4
No	1
Work Status	
On maternity leave	2
Employed full-time	3
Employed part-time/casual	12
Home duties/unemployed	7
Student	4
SEIFA ^2^ using Tertiles	
High SEIFA	17
Mid SEIFA	6
Low SEIFA	5
Mean Child Age (months) ± SD	38.68 ± 11.1 or (3.22 ± 0.93) years

^1^ TAFE: vocational education for apprenticeships and traineeships. ^2^ SEIFA: socio-economic indexes for areas, a measure developed by the Australian Bureau of Statistics that ranks geographic areas in Australia according to relative socio-economic advantage and disadvantage [38].

**Table 2:** The six main categories of constructs elicited from the mothers.

Construct Categories	Number of Times the Participants Used the Construct
**Health and Well-being**	41
e.g., Unhealthy/healthy	
*Hyperactivity*	
e.g., No effect on behaviour/can affect behaviour	
*Nutrition*	
e.g., Less nutrition for children/more nutrition for children;	
Contains no protein/contains protein	
*Satiety/Digestion*	
e.g., Does not keep him full/keeps him full	
*Teeth*	
e.g., Bad for teeth/better for teeth	
**Sugar—natural and artificial, intrinsic and extrinsic**	33
e.g., Contains no sugar/contains sugar;	
Natural sugar/artificial sugar;	
**Dairy**	28
e.g., Not milk-based/milk-based;	
No calcium/contains calcium	
**Packaging**	25
e.g., Not a plastic bottle/plastic bottle	
One time open/resealable	
**Additives**	22
e.g., Close to nature/contains additives	
**Pure, natural/man-made, artificial**	19
e.g., No or few natural ingredients/contains natural ingredients	
**Preparation**	19
e.g., Not ready-made/ready made	
**Fruit juice, fruit-based, made from real fruit**	17
e.g., Fruit-based/not fruit-based	
**Carbonation**	15
e.g., Not-carbonated/carbonated	
**Flavouring**	14
e.g., Natural flavour/artificial flavour	
**Soft Drink**	11
e.g., Not a soft-drink/soft drink	
**Diet Claims**	9
e.g., non diet/diet	
**Targeted to Children**	9
e.g., Not tailored to children/tailored to children	
**Real/Processed**	8
e.g., Not processed/processed	
**Miscellaneous**	8
e.g., Weak brand/strong brand	
Plant-based/animal-based	
Less tasty/more tasty	
Single serve/multiple serves	
**Water**	7
e.g., Little water concentration/essentially water	
**Caffeine**	6
e.g., No caffeine/contains caffeine	
**Organic**	6
e.g., Not organic/organic	
**Appearance**	5
e.g., Clear-opaque	
**Context**	4
e.g., Not a breakfast drink/breakfast drink	
Not a night-time drink-night time drink	
**Convenience/Cost Value**	4
e.g., Free, not-bought/must be purchased	
Have at home/have to buy	
**Origin/Environment**	3
e.g., Made in Australia/overseas owned	
Not environmentally friendly/environmentally friendly	
**Total**	312
